# TGF-β1 sensitizes TRPV1 through Cdk5 signaling in odontoblast-like cells

**DOI:** 10.1186/1744-8069-9-24

**Published:** 2013-05-13

**Authors:** Elias Utreras, Michaela Prochazkova, Anita Terse, Jacklyn Gross, Jason Keller, Michael J Iadarola, Ashok B Kulkarni

**Affiliations:** 1 Functional Genomics Section, Laboratory of Cell and Developmental Biology, National Institute of Dental and Craniofacial Research, National Institutes of Health, 30 Convent Drive, Building 30, Room 130, Bethesda, MD 20892, USA; 2 Laboratory of Cellular and Neuronal Dynamics, Faculty of Science, University of Chile, Santiago, Chile; 3 Neurobiology and Pain Therapeutics Section, National Institute of Dental and Craniofacial Research, National Institutes of Health, Bethesda, MD, 20892, USA

**Keywords:** TGF-β1, Cdk5, p35, TRPV1, MDPC-23 cells

## Abstract

**Background:**

Odontoblasts are specialized cells that form dentin and they are believed to be sensors for tooth pain. Transforming growth factor-β1 (TGF-β1), a pro-inflammatory cytokine expressed early in odontoblasts, plays an important role in the immune response during tooth inflammation and infection. TGF-β1 is also known to participate in pain signaling by regulating cyclin-dependent kinase 5 (Cdk5) in nociceptive neurons of the trigeminal and dorsal root ganglia. However, the precise role of TGF-β1 in tooth pain signaling is not well characterized. The aim of our present study was to determine whether or not in odontoblasts Cdk5 is functionally active, if it is regulated by TGF-β1, and if it affects the downstream pain receptor, transient receptor potential vanilloid-1 (TRPV1).

**Results:**

We first determined that Cdk5 and p35 are indeed expressed in an odontoblast-enriched primary preparation from murine teeth. For the subsequent analysis, we used an odontoblast-like cell line (MDPC-23) and found that Cdk5 is functionally active in these cells and its kinase activity is upregulated during cell differentiation. We found that TGF-β1 treatment potentiated Cdk5 kinase activity in undifferentiated MDPC-23 cells. SB431542, a specific inhibitor of TGF-β1 receptor 1 (Tgfbr1), when co-administered with TGF-β1, blocked the induction of Cdk5 activity. TGF-β1 treatment also activated the ERK1/2 signaling pathway, causing an increase in early growth response-1 (Egr-1), a transcription factor that induces p35 expression. In MDPC-23 cells transfected with TRPV1, Cdk5-mediated phosphorylation of TRPV1 at threonine-407 was significantly increased after TGF-β1 treatment. In contrast, SB431542 co-treatment blocked TRPV1 phosphorylation. Moreover, TGF-β1 treatment enhanced both proton- and capsaicin-induced Ca^2+^ influx in TRPV1-expressing MDPC-23 cells, while co-treatment with either SB431542 or roscovitine blocked this effect.

**Conclusions:**

Cdk5 and p35 are expressed in a murine odontoblast-enriched primary preparation of cells from teeth. Cdk5 is also functionally active in odontoblast-like MDPC-23 cells. TGF-β1 sensitizes TRPV1 through Cdk5 signaling in MDPC-23 cells, suggesting the direct involvement of odontoblasts and Cdk5 in dental nociceptive pain transduction.

## Background

Odontoblasts, the polarized columnar cells localized at the periphery of the dental pulp, synthesize and secrete collagenous and non-collagenous matrix proteins, such as dentin sialophosphoprotein (DSPP), during dentinogenesis to form dentin [1]. Many growth factors, such as transforming growth factor-β (TGF-β), fibroblast growth factors (FGFs), and insulin-like growth factors (IGFs), are believed to be mediators of the epithelial-mesenchymale interactions involved in the functional differentiation of odontoblasts [2]. In particular, TGF-β1, a prototype member of the TGF-β superfamily, is expressed in a wide variety of developing tissues from the earliest stages. TGF-β1 is also expressed in odontoblasts and ameloblasts during the early stages of tooth development [3]. We previously identified an important role for TGF-β signaling in the mineralization and formation of dentin in mice over-expressing TGF-β1 specifically in tooth [4]. We also discovered that altered TGF-β1 expression in tooth impacts the adhesion process of ameloblasts [5]. Interestingly, various studies on odontoblast-like MDPC-23 cells also revealed vital roles for active TGF-β signaling in the regulation of DSPP expression [6-8] and in cell migration through activation of the p38 MAPK and AKT signaling pathways [6].

However, the impact of TGF-β signaling on tooth pain is far from clear. Tooth pain is mainly characterized by the exposure of dentin to direct mechanical, chemical, and/or thermal stimulation. Recent reports indicate that odontoblasts express various family members of the transient receptor potential (TRP) ion channels, such as TRPV1, TRPV2, TRPV3, TRPV4, TRPA1, TRPM3, and TRPM8. TRP channels are believed to participate in the underlying molecular mechanisms involved in thermal and mechanical sensory transduction [1,9-12]. Furthermore, in functional assays using either cultured odontoblast-like cells or native human odontoblasts, specific agonists of either TRPV1, TRPA1, or TRPM8 elicited channel activation and transient influxes of Ca^2+^ that could be blocked by their respective antagonists [11,13].

We previously discovered that cyclin-dependent kinase 5 (Cdk5), a proline-directed serine/threonine kinase, plays a pivotal role in inflammatory pain [14-18]. Cdk5 kinase activity is predominant in post-mitotic neurons where its activators, p35 and p39, are expressed, although recently Cdk5 activity has also been detected in non-neuronal tissues [19,20]. Increased expression of p35, which occurred after experimentally-induced inflammation, was associated with elevated Cdk5 activity in rat nociceptive primary afferent neurons [18]. We also identified that Cdk5-mediated phosphorylation of TRPV1 at Thr407 is involved in thermal nociception and inflammatory pain [21]. We have further demonstrated that tumor necrosis factor-α (TNF-α) increases Cdk5 activity [16,17], while resveratrol, a polyphenolic compound with known analgesic activity, inhibits Cdk5 activity [14]. Most importantly, we recently discovered that TGF-β1 is a key regulator of Cdk5 activity in nociceptive neurons, indicating that active crosstalk between the TGF-β1 and Cdk5 pathways plays an important role in inflammation-induced pain signaling [15]. However, the role of similar crosstalk between TGF-β and Cdk5 has not been studied in relation to tooth pain, which is frequently induced by inflammation associated with an infection that affects odontoblast cells. Therefore, in the current study, we have evaluated the expression of Cdk5 and p35 in both an odontoblast-enriched preparation from murine teeth and in the odontoblast-like MDPC-23 cell line. To evaluate its possible involvement in tooth pain, we then characterized the regulation of Cdk5 activity by TGF-β1, detected the Cdk5-mediated phosphorylation of TRPV1, and finally measured its effect on proton- and capsaicin-induced TRPV1 activation.

## Results

### Expression of Cdk5 and p35 in an odontoblast-enriched preparation from murine teeth

**Figure 1:**
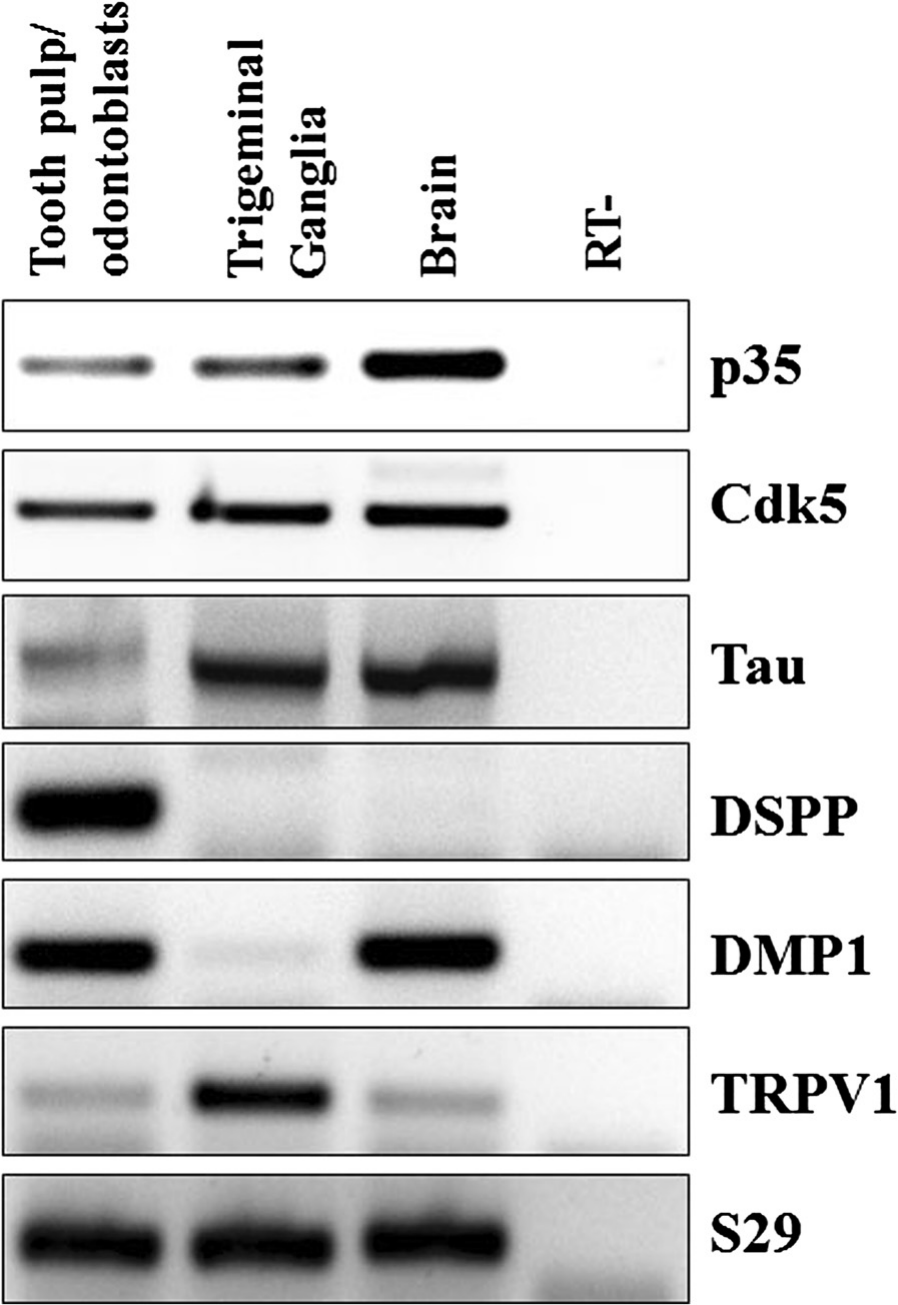
**Expression of Cdk5 and p35 in an odontoblast-enriched preparation from murine teeth.** Representative RT-PCR analysis of Cdk5 and p35 mRNA levels in an odontoblast-enriched preparation from murine teeth, TG and brain tissue. DSPP and DMP1 mRNA expression were used as odontoblast markers. Tau was used as a neuronal marker and TRPV1 was used as a nociceptive neuron marker. S29 was used as a housekeeping gene. RT- indicates negative control.

To determine whether mouse odontoblasts express Cdk5 and p35, we extracted total RNA from an odontoblast-enriched primary preparation from mouse incisors and performed RT-PCR analysis. As a positive control for Cdk5 and p35 expression, we used mouse brain and trigeminal ganglion (TG). We found that Cdk5 and p35 mRNAs are expressed in the odontoblast-enriched preparation at levels similar to TG, but less compared to brain (Figure 1). To evaluate the purity of our odontoblast-enriched preparation, we analyzed the expression of dentin sialophosphoprotein (DSPP) and dentin matrix acidic phosphoprotein 1 (DMP1), two well-characterized odontoblast markers. Interestingly, we found DSPP expression only in the odontoblast-enriched preparation, whereas DMP1 was found not only in our odontoblast-enriched preparation but also in brain, confirming an earlier report [22]. Moreover, we detected the expression of Tau, a neuronal marker, in our odontoblast-enriched preparation. Interestingly, we found detectable expression of TRPV1 in our odontoblast-enriched preparation, but it was less robust as compared to TG. Taken together, these results confirm the expression of Cdk5 and p35, as well as TRPV1, in our odontoblast-enriched preparation from mouse incisors, implicating a potential role for Cdk5 in tooth pain.

### Differentiation of MDPC-23 cells induces activation of the TGF-β signaling pathway

**Figure 2:**
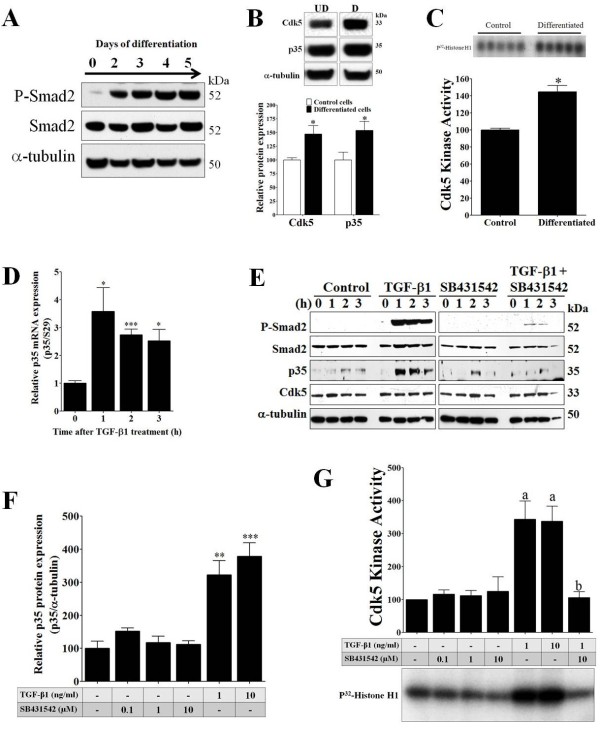
**Differentiation or TGF-β1 treatment increases p35 expression and Cdk5 activity in MDPC-23 cells. A**, Representative Western blot analysis showing activation of the TGF-β1 signaling pathway, determined by an increase of phospho-Smad2, on successive days during MDPC-23 cell differentiation. **B**, Upper panel shows a representative Western blot analysis of Cdk5 and p35 protein levels in undifferentiated (UD) and 5-day differentiated (D) MDPC-23 cells. Lower panel shows quantification of the Western blot for Cdk5 and p35 in control cells and differentiated MDPC-23 cells. **C**, Cdk5 kinase activity was measured from immunoprecipitates of control and 5-day differentiated MDPC-23 cells. **D**, q-PCR analysis of p35 mRNA levels normalized against S29. Total RNA was obtained from MDPC-23 cells treated with TGF-β1 (1 ng/ml) for 0, 1, 2, and 3 h. **E**, Western blot analysis of phospho-Smad2, Smad2, p35, Cdk5, and α-tubulin was performed in MDPC-23 cells treated with: vehicle (control), TGF-β1 (1 ng/ml), SB431542 (10 μM), and TGF-β1 (1 ng/ml) plus SB431542 (10 μM) over the course of 0, 1, 2, and 3 h. **F**, Quantification of Western blot of p35 and α-tubulin was performed in MDPC-23 cells treated with: vehicle (control), TGF-β1 (1 ng/ml), SB431542 (10 μM) over 24 h. **G**, Cdk5 kinase activity measured from immunoprecipitates of MDPC-23 cells treated with vehicle (control), SB431542 (0.1, 1, and 10 μM), TGF-β1 (1 and 10 ng/ml), and TGF-β1 (1 ng/ml) plus SB431542 (10 μM) over 24 h. All data are presented as the mean and SEM (*n* = 3-5). * *p <* 0.05, ** p *<*0.01; *** p *<*0.001 (t-Test); a: *p <* 0.01 TGF-β1 treatment vs. control; and b: *p <* 0.01 TGF-β1 vs. TGF-β1 plus SB431542 treatment (Bonferroni’s test after ANOVA).

We previously reported that Cdk5 is a key player in pain signaling [17,18]. TNF-α and TGF-β1 regulate Cdk5 activity in sensory neurons by modulating the expression of p35, a co-activator of Cdk5 [15,16]. To investigate whether Cdk5 plays a similar role in tooth pain signaling, we examined its expression and kinase activity in MDPC-23 cells, an odontoblast-like cell line derived from rodent dental papilla cells [23-25]. MDPC-23 cells can be induced to differentiate by the addition of ascorbate (50 μg/ml) and β-glycerophosphate (10 mM) to the culture medium. We analyzed the differentiation process of MDPC-23 cells by observing cell morphology under a microscope; we performed this daily for 5 days after starting the treatment with ascorbate and β-glycerophosphate. We observed the formation of multilayered nodules with multiple cell membrane processes (data not shown), similar to the phenotype described by others [23]. Moreover, it is also known that TGF-β1 regulates this differentiation process [7]. We therefore evaluated the activation of the TGF-β signaling pathway in MDPC-23 cells over 5 consecutive days of induced differentiation, using Western blot analysis with antibodies directed against phospho-Smad2 (Ser465/467) and total Smad2 (Figure 2A). We found that, as early as 2 days, phospho-Smad2 levels increased significantly. Moreover, phospho-Smad2 levels then remained elevated through the 5 day course (Figure 2A).

### Differentiation of MDPC-23 cells induces expression of Cdk5 and p35, with a subsequent increase in Cdk5 kinase activity

In order to address whether Cdk5 and p35 are expressed in MDPC-23 cells, we conducted qPCR on total RNA isolated from undifferentiated MDPC-23 cells and from PC12 cells, a positive control for Cdk5 and p35 expression [16]. We found that Cdk5 and p35 mRNAs were expressed in MDPC-23 cells at similar levels compared to PC12 cells (data not shown). We then investigated whether differentiation of MDPC-23 cells regulates Cdk5 and p35 expression. After 5 days of induced differentiation, Cdk5 and p35 protein levels were analyzed by Western blot analysis. We found that Cdk5 and p35 protein levels were significantly increased in differentiated MDPC-23 cells as compared to undifferentiated MDPC-23 cells (Figure 2B). Since the p35 protein level is a limiting factor for Cdk5 kinase activity [26], we analyzed whether the differentiation-mediated increase in p35 expression results in an increase of Cdk5 activity. We immunoprecipitated Cdk5 protein from the undifferentiated and differentiated MDPC-23 cells using a Cdk5 antibody, and we then assayed Cdk5 kinase activity by using histone H1 as a substrate. We found that Cdk5 kinase activity was significantly increased in differentiated versus undifferentiated MDPC-23 cells (Figure 2C).

### TGF-β1 treatment increases p35 protein levels and Cdk5 kinase activity in MDPC-23 cells

We previously determined that TGF-β1 can regulate Cdk5 kinase activity in sensory neurons through an increase in p35 expression [15]. To evaluate whether the activation of the TGF-β signaling pathway during the differentiation process affects Cdk5 kinase activity in MDPC-23 cells, we examined the effects of recombinant TGF-β1 treatment on p35 expression and Cdk5 kinase activity in undifferentiated MDPC-23 cells. We deprived MDPC-23 cells of serum for 1 h and then treated these cells with either vehicle (control), TGF-β1 (1 ng/ml), Tgfbr1 inhibitor (SB431542, 10 μM), or TGF-β1 (1 ng/ml) plus SB431542 (10 μM) for 0, 1, 2 and 3 h. We found that 1-3 h of TGF-β1 treatment resulted in a significant increase of phospho-Smad2 (Ser465/467) levels. In contrast, this effect was blocked in cells treated either with SB431542 alone or TGF-β1 plus SB431542 (Figure 2E). Most importantly, TGF-β1 treatment significantly increased p35 mRNA levels as early as 1 h after treatment and they remained elevated after 3 h of treatment as determined by qPCR (Figure 2D). However, Cdk5 mRNA levels were unchanged at each time point evaluated (data not shown). Interestingly, p35 protein levels were also significantly increased after 1 h of TGF-β1 treatment and remained high at 3 h (Figure 2E) and 24 h (Figure 2F). Cdk5 protein levels did not change after 0–3 h of TGF-β1 treatment (Figure 2E). In contrast, SB431542 treatment with or without TGF-β1 totally blocked the increase of p35 protein levels (Figure 2E), suggesting that activation of the TGF-β signaling pathway (via phosphorylation of Smad2) is essential for regulating p35 expression in MDPC-23 cells.

**Figure 3:**
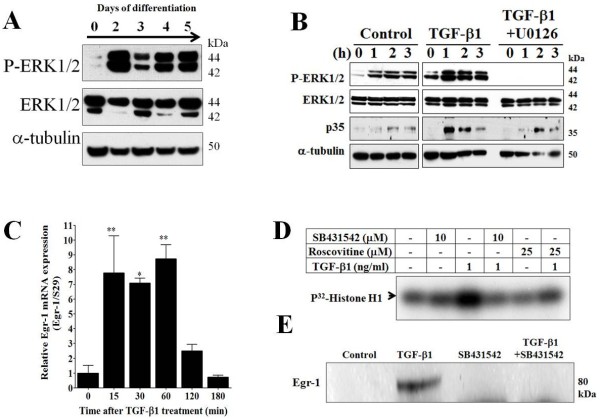
**Differentiation or TGF-β1 treatment induces activation of the ERK1/2 pathway and increases Cdk5 activity in MDPC-23 cells. A**, Representative Western blot analysis showing activation of ERK1/2 signaling pathways measured by an increase in phospho-ERK1/2 on successive days during differentiation of MDPC-23 cells. **B**, Representative Western blot analysis of phospho-ERK1/2, ERK1/2, p35, and α-tubulin was performed in MDPC-23 cells treated with: vehicle (control), TGF-β1 (1 ng/ml), and TGF-β1 (1 ng/ml) plus U0126 (20 μM) over the course of 0, 1, 2, and 3 h. **C**, qPCR analysis of Egr-1 mRNA levels normalized against S29. Total RNA was obtained from MDPC-23 cells treated with TGF-β1 (1 ng/ml) for 0, 15, 30, 60, 120, and 180 min. **D**, The effect of roscovitine on Cdk5 kinase activity. MDPC-23 cells were treated with vehicle (control), SB431542 (10 μM), TGF-β1 (1 ng/ml), TGF-β1 (1 ng/ml) plus SB431542 (10 μM), roscovitine (25 μM), and TGF-β1 (1 ng/ml) plus roscovitine (25 μM) over 24 h. **E**, Western blot analysis against Egr-1 from MDPC-23 cells treated with: vehicle (control), TGF-β1 (1 ng/ml), SB431542 (10 μM), and TGF-β1 (1 ng/ml) plus SB431542 (10 μM) over 24 h. All data are presented as the mean and SEM (*n* = 4). *p < 0.05, **p < 0.01, (Dunnett’s test after ANOVA).

Since p35 expression was induced by TGF-β1 treatment, we evaluated whether Cdk5 kinase activity was also affected in MDPC-23 cells. We found significantly increased Cdk5 kinase activity after 24 h of TGF-β1 treatment (1 and 10 ng/ml) (Figure 2G). In contrast, SB431542 treatment (0.1, 1, and 10 μM) did not alter basal Cdk5 kinase activity; however, co-treatment with TGF-β1 (1 ng/ml plus 10 μM SB431542) significantly blocked the TGF-β1-mediated increase of Cdk5 kinase activity (Figure 2G). Additionally, we analyzed whether roscovitine, a Cdk5 inhibitor, would inhibit the Cdk5 kinase activity induced by TGF-β1 in MDPC-23 cells. We found that basal Cdk5 kinase activity decreased in the presence of roscovitine (25 μM) and, during co-treatment with TGF-β1 (1 ng/ml), that roscovitine blocked the TGF-β1-mediated increase of Cdk5 kinase activity (Figure 3D).

### Differentiation of MDPC-23 cells induces activation of the ERK1/2 signaling pathway

It has been reported that several compounds, such as sodium fluoride (NaF) [27], amelogenin [28], or lipopolysaccharide (LPS) [29], can activate the ERK1/2 signaling pathway in MDPC-23 cells. The activation of the ERK1/2 signaling pathway was found to be crucial for regulating p35 expression [14-16,30], and it has also been reported that TGF-β1 can activate non-Smad signaling pathways such as the ERK1/2 pathway [31]. Therefore, we evaluated whether the ERK1/2 signaling pathway is affected during the MDPC-23 differentiation process. We found that levels of phospho-ERK1/2 (Thr202/Tyr204) were increased after 2 days of differentiation and remained elevated at each subsequent time point they were measured, while total ERK1/2 protein levels did not change (Figure 3A). To evaluate whether TGF-β1 affects activation of the ERK1/2 signaling pathway, MDPC-23 undifferentiated cells were pre-treated with a specific MEK1 inhibitor, U0126 (20 μM), 30 min before adding TGF-β1 (1 ng/ml) for 0 to 3 h (Figure 3B). TGF-β1 increased phospho-ERK1/2 levels at 1 to 3 h, and also increased p35 protein levels at these time points when compared to control cells. In contrast, when cells were co-treated with U0126 (20 μM) and TGF-β1 (1 ng/ml), there was complete inhibition of phospho-ERK1/2 at 0 to 3 h. U0126 inhibited the p35 protein induction caused by 1 h of TGF-β1 treatment but not after 2 or 3 h, where p35 protein levels increased similar to cells receiving only TGF-β1 (Figure 3B). This suggests that the increase in p35 protein, which we found in MDPC-23 cells to be mediated by TGF-β1, is in part dependent upon activation of the ERK1/2 signaling pathway but likely also upon other independent mechanisms as well.

We and others have reported that early growth response-1 (Egr-1), an important transcription factor that regulates p35 expression, is rapidly upregulated after NGF or TNF-α treatment in PC12 cells [14,16,30], and after TGF-β1 treatment in B104 rat neuroblastoma cells [15]. Here, we evaluated whether Egr-1 mRNA expression is regulated by TGF-β1 treatment in undifferentiated MDPC-23 cells by using qPCR. We found that TGF-β1 (1 ng/ml) treatment produced a significant increase in Egr-1 mRNA levels after 15 min, and these levels remained elevated until 1 h but then decreased to basal levels at 2 and 3 h (Figure 3C). Additionally, we found that Egr-1 protein levels increased after 24 h of TGF-β1 treatment (1 ng/ml), while co-treatment with SB431542 (10 μM) or SB431542 alone blocked the increase in Egr-1 protein levels (Figure 3E). Collectively, these results suggest that TGF-β1 induces a sustained and robust increase in p35 levels, possibly through increased Egr-1 expression.

### TGF-β1 increases Cdk5-mediated phosphorylation of TRPV1 and potentiates intracellular Ca^2+^ influx in MDPC-23 cells

**Figure 4:**
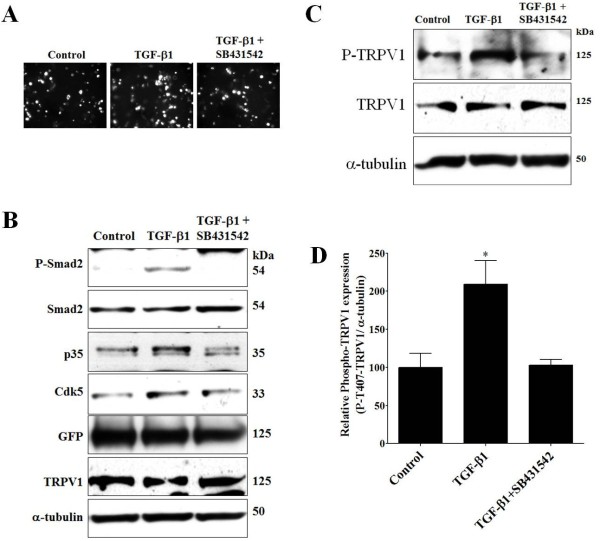
**TGF-β1 potentiates Cdk5-mediated phosphorylation of TRPV1 in MDPC-23 cells. A**, Representative fluorescence of MDPC-23 cells transiently transfected with the TRPV1-GFP plasmid vector after 48 h. After 24 h of transfection, MDPC-23 cells were treated with vehicle (control), TGF-β1 (1 ng/ml), and TGF-β1 (1 ng/ml) plus SB431542 (10 μM), and protein extracts were analyzed by Western blot against phospho-Thr407-TRPV1, TRPV1, and α-tubulin 24 h later. **B**, Representative Western blot analysis showing phospho-Smad2, Smad2, p35, Cdk5, GFP, TRPV1, and α-tubulin from MDPC-23 cells treated with vehicle (control), TGF-β1 (1 ng/ml), and TGF-β1 (1 ng/ml) plus SB431542 (10 μM) over 24 h. **C**, Representative Western blot analysis against phospho-Thr407-TRPV1 in MDPC-23 cells treated with vehicle (control), TGF-β1 (1 ng/ml), and TGF-β1 (1 ng/ml) plus SB431542 (10 μM) over 24 h. **D**, Quantification analysis showed that TGF-β1 treatment significantly increased phospho-Thr407-TRPV1 compared to control, and SB431542 blocked this effect. All data are presented as the mean and SEM (*n* = 3). *p < 0.05, (t-Test).

Odontoblast cells have been linked to dental nociception [1,9,12,32] due to the expression of functional TRPV1 channels found in these cells from human [11] and mouse [10]. We previously reported that Cdk5 can phosphorylate TRPV1, specifically at Thr407 [21]. Therefore, we evaluated whether a TGF-β1-mediated increase in Cdk5 activity could regulate phosphorylation of TRPV1 at Thr407 in MDPC-23 cells. The expression of TRPV1 has not been previously reported in MDPC-23 cells, and we measured TRPV1 protein levels using Western blot analysis but could not find detectable TRPV1 protein (data not shown). Therefore, we transiently transfected MDPC-23 cells with a CMV promoter-driven TRPV1-GFP vector [33]. After 24 h of transfection, MDPC-23 cells were treated with either vehicle (control), TGF-β1 (1 ng/ml), or TGF-β1 (1 ng/ml) plus SB431542 (10 μM), and protein extracts were analyzed by Western blotting. We found the amount of GFP fluorescence in transfected MDPC-23 cells remained similar after all three treatments (Figure 4A). In addition, we evaluated the activation of TGF-β1 signaling (phospho-Smad2 and total Smad2 protein levels) and the expression of p35, Cdk5, GFP, TRPV1, and α-tubulin after all three treatments using Western blot analysis. We found that TGF-β1 treatment increased phospho-Smad2 and p35 protein levels, and this increase was blocked in cells co-treated with SB431542 (Figure 4B). Interestingly, GFP and TRPV1 levels remained equivalent in all three treatment groups (Figure 4B). Most importantly, we found that TGF-β1 treatment significantly increased phospho-Thr407-TRPV1 levels when compared to control cells, while phosphorylation of TRPV1 was blocked in MDPC-23 cells co-treated with SB431542 (Figure 4C and D). These results suggest that in TRPV1-expressing MDPC-23 cells, p35 protein levels increase in response to TGF-β1, resulting in elevated Cdk5 activity and TRPV1 phosphorylation.

**Figure 5:**
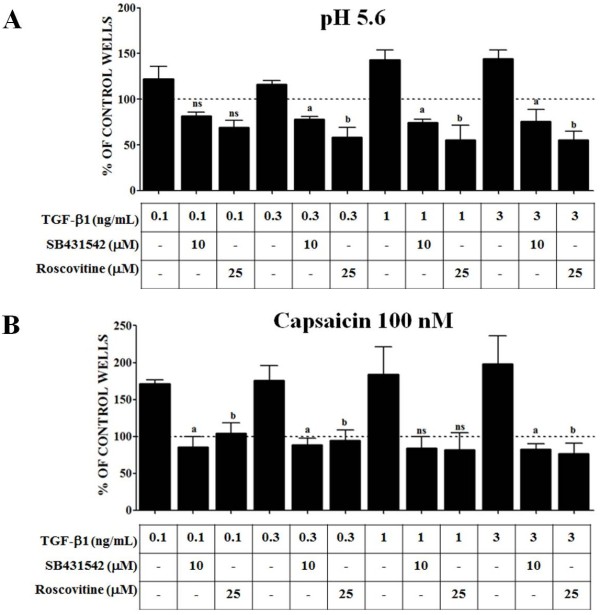
**TGF-β1 potentiates Cdk5-mediated phosphorylation of TRPV1, resulting in increased intracellular Ca**^**2+ **^**influx in MDPC-23 cells. **MDPC-23 cells stably transfected with rat TRPV1 were treated with ascending concentrations of TGF-β1 (0.1, 0.3, 1, and 3 ng/ml), with or without SB431542 (10 μM) or roscovitine (25 μM) over 24 h. We then examined both proton- and capsaicin-induced calcium influx in these cells. Ca^2+ ^influx was stimulated with a pH 5.6 buffer (**A**) or with 100 nM capsaicin (**B**). The total influx of calcium under both conditions increased significantly following TGF-β1 treatment. In contrast, co-treatment with SB431542 or roscovitine produced a significant decrease in calcium influx caused by the low pH buffer or by capsaicin. All data are presented as the mean and SEM (*n* = 3). a: *p <* 0.05 TGF-β1 vs. TGF-β1 plus SB431542 treatment; and b: *p <* 0.05 TGF-β1 vs. TGF-β1 plus roscovitine treatment (t-Test). ns: not significant.

To evaluate whether increased TRPV1 phosphorylation in MDPC-23 cells treated with TGF-β1 has a physiological effect, we examined proton- and capsaicin-induced calcium influx in these cells (Figure 5A and B, respectively). Ca^2+^ influx was measured in MDPC-23 cells stably transfected with rat TRPV1 cDNA, and then these cells were activated either with low pH buffer (Figure 5A) or with 100 nM capsaicin (Figure 5B) in the presence of calcium-45 (^45^Ca^+2^). Confluent cells were pre-treated with TGF-β1 (0.1 to 3.0 ng/ml) alone, TGF-β1 in the presence of SB431542 (10 μM), or TGF-β1 in the presence of roscovitine (25 μM), then cells were assayed for calcium uptake at 24 h. We found enhanced calcium uptake by cells treated with TGF-β1, compared to untreated control cells (dashed line at “100%” in Figure 5A and 5B), and this effect was blocked when cells were co-treated with either roscovitine or SB431542. Therefore, these results suggest that TGF-β1-mediated phosphorylation of TRPV1 potentiates proton- and capsaicin-induced Ca^2+^ influx in TRPV1-expressing MDPC-23 cells.

## Discussion

We posed three main questions in this study: 1) Do odontoblasts and the odontoblast-like MDPC-23 cells express functional Cdk5/p35; 2) If yes, is there a crosstalk between Cdk5 and the TGF-β signaling pathway; 3) Does the crosstalk have an impact on nociceptors, specifically TRPV1. These three questions are crucial for determining the potential role of Cdk5/p35 in nociception and pain transduction by odontoblasts. Our results clearly demonstrate that Cdk5 and p35 are expressed in an odontoblast-enriched preparation from murine teeth as well as in the odontoblast-like MDPC-23 cell line. We found that Cdk5 kinase is active in MDPC-23 cells. In addition, Cdk5 and p35 protein levels, and Cdk5 kinase activity, increased in MDPC-23 cells during differentiation. Interestingly, the TGF-β and ERK1/2 signaling pathways were activated during the differentiation process, suggesting that Cdk5 activity is regulated by TGF-β1 and ERK1/2 in these cells. Furthermore, we found that TGF-β1 treatment of MDPC-23 cells increased the mRNA and protein levels of p35, resulting in a subsequent increase in Cdk5 kinase activity. A Tgfbr1 inhibitor, SB431542, blocked this effect. We also found that Cdk5-mediated phosphorylation of TRPV1 was significantly increased by TGF-β1 treatment, while co-treatment with SB431542 again blocked this effect. TGF-β1 treatment potentiated proton- and capsaicin-induced Ca^+2^ influx in MDPC-23 cells stably transfected with TRPV1, while SB431542 and roscovitine inhibited this effect. Collectively, our results indicate that Cdk5/p35 may play an important role in odontoblast function, specifically in relation to nociception.

Odontoblasts form a layer of specialized cells localized directly beneath dentin, separating the dentin from tooth pulp. Because of the morphological shape of odontoblasts, they are believed to play a pivotal role in nociception. Odontoblast cells have a cellular process that extends into a liquid phase (dentinal fluid) in calcified tubules. Thus, odontoblasts are able to sense both external stimuli and transient changes in the pulp microcirculation [32]. But currently, there are three prevailing theories regarding the mechanism underlying dental nociception: 1) neural, 2) hydrodynamic, or 3) odontoblastic. Of the three, the hydrodynamic theory is the most widely accepted [34]. However, the odontoblastic mechanism is gaining attention due to a recent finding on the capacity of these cells to generate action potentials [35], and to their functional expression of several family members of the TRP ion channels [1,9-13,32,36], as well as the TREK-1 channel [37]. In addition, dental pulp expresses both ATP receptors [38] and ecto-ATPase NTPDase2, one of the principal enzymes responsible for extracellular ATP hydrolysis, suggesting the presence of an apparatus for ATP release and degradation in human dental pulp [39]. Our findings on the expression of Cdk5 and p35 in odontoblast-like cells, and on the regulation of Cdk5 kinase activity by TGF-β1, support the theory that odontoblasts are directly involved in dental nociception and pain transduction.

We and others have delineated a role for Cdk5 in sensory neurons during inflammatory hyperalgesia [14-18,21,40-44]. Cdk5 has also been shown to be involved in trigeminal neuropathic pain [45]. However, there are no studies describing the expression or function of Cdk5 in odontoblast cells. Several functions have already been described for Cdk5 and p35 in non-neuronal cells [19,20,46]. We demonstrate here for the first time that Cdk5 and p35 are expressed in an odontoblast-enriched extract from murine teeth as well as in odontoblast-like MDPC-23 cells. Moreover, TGF-β1 treatment increases Cdk5 kinase activity in MDPC-23 cells, suggesting that Cdk5/p35 might participate in several functions, but particularly in nociception. We previously demonstrated a key function for TGF-β1 during odontoblast differentiation, where it down regulates DSPP expression in mice that over-express TGF-β1 specifically in teeth [4]. Likewise, we found that TGF-β1 also participates in tooth mineralization, impacting the adhesion of ameloblasts to dentin [5]. Moreover, TGF-β1 activates the Smad3 signaling pathway to down regulate DSP [7] and is important during migration of odontoblast-like MDPC-23 cells [6]. TGF-β1 has also been associated with facial pain, since TGF-β1 levels were found to be significantly elevated in the plasma and cerebrospinal fluid of migraineurs [47,48]. Most importantly, we recently discovered that mice deficient in TGF-β1 signaling have decreased Cdk5 kinase activity and diminished TRPV1 phosphorylation in the trigeminal and dorsal root ganglia, suggesting that an active crosstalk between the TGF-β1 and Cdk5 signaling pathways impacts peripheral inflammatory pain [15]. Here, we have identified potential involvement of TGF-β1 and Cdk5 in dental nociception.

**Figure 6:**
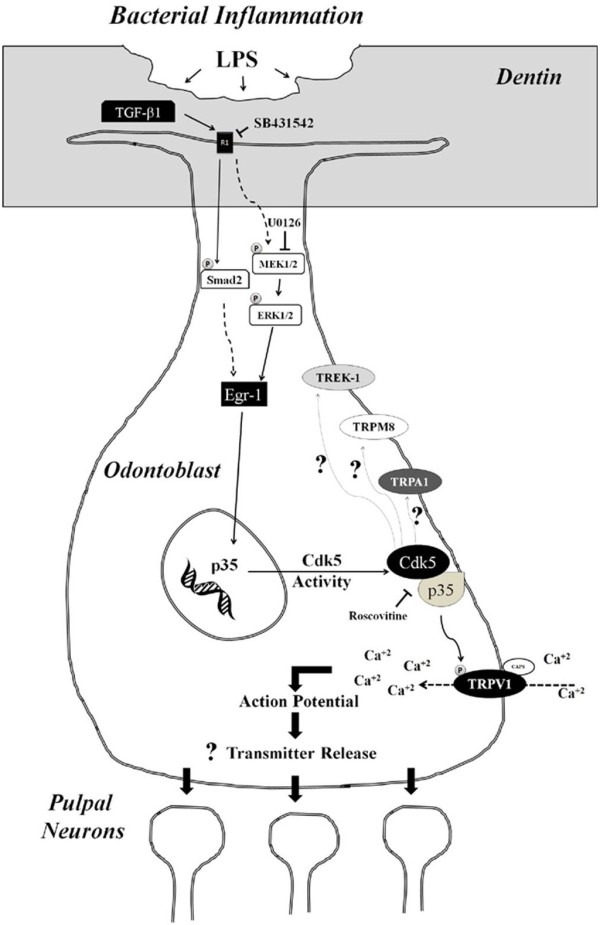
**Proposed model illustrating the participation of Cdk5 in signaling pathways that influence dental nociception. **Bacterial inflammation occurring in dental caries increases secretion of TGF-β1 into the extracellular matrix, which in turn activates Smad dependent and independent pathways in odontoblast cells. These cascades activate Cdk5 activity by increasing Egr-1 and p35 expression. Then, the Cdk5/p35 complex phosphorylates TRPV1, resulting in enhanced calcium influx and the subsequent activation of action potentials, which in turn may increase transmitter release. Pain transmission would occur through transmitter-mediated activation of pulpal neurons. Moreover, Cdk5 may modulate the sensitivity of other nociceptors such as TRPA1, TRPM8, or TREK-1 during inflammation-induced tooth pain.

There is accumulating body of evidence that supports our findings. From one study, the number of TGF-β1 positive cells was significantly increased during pulpitis in the human odontoblast layer (ODL) [49,50]. Another report showed that several cytokines, chemokines, and their receptors, were upregulated in human ODL during tooth caries [51], which are essentially caused by bacteria and yeast that colonize dentin and root cementum [52]. Additionally, it was shown that immunoreactivity for TGF-β1 was significantly increased in the odontoblast and pulpal cells of carious teeth [53]. These findings indicate that TGF-β1 is upregulated in normal pathological conditions, such as carious inflammation, further suggesting that TGF-β1 is vital not only in resolving inflammation and promoting wound healing, but also likely involved in pain signaling. Based on our studies, we propose a model where TGF-β1 is secreted during bacterial inflammation and promotes Cdk5 kinase activity in odontoblasts. This in turn leads to the phosphorylation of TRPV1, other TRP ion channels, or TREK-1 channels, which then potentiates calcium influx during stimulation. The subsequent depolarization caused by calcium influx activates downstream effectors, possibly leading to transmitter release from odontoblasts and pain signal transmission (Figure 6).

## Conclusions

Primary odontoblasts and odontoblast-like MDPC-23 cells express functional Cdk5/p35. TGF-β1 treatment increases Cdk5 activity in TRPV1-expressing MDPC-23 cells. This subsequently leads to increased TRPV1 phosphorylation, thereby potentiating proton- and capsaicin-induced calcium influx in these cells. Odontoblasts are therefore suggested to be directly involved in dental nociception, which can be modulated by Cdk5.

## Methods

### Materials

Tgfbr1 inhibitor (SB431542), DMSO, ascorbate, β-glycerophosphate, histone H1, roscovitine, and α-tubulin antibody were obtained from Sigma (St. Louis, MO). MEK1 inhibitor (U0126) was obtained from Cell Signaling Technology (Beverly, MA). Recombinant TGF-β1 was obtained from R&D Systems (Minneapolis, MN). Protein quantification reagents were obtained from Bio-Rad Laboratories (Hercules, CA), and enhanced chemiluminescence reagents for Western blot analysis were purchased from Thermo Scientific (Rockford, IL).

### Antibodies

Antibodies to Cdk5, p35, Egr-1, TRPV1, and secondary antibodies (HRP-conjugated goat anti-mouse, anti-rabbit antibodies) were obtained from Santa Cruz Biotechnology, Inc. (Santa Cruz, CA). Anti-phospho-Thr407-TRPV1 antibodies were generated previously [21]. Antibodies to phospho-ERK1/2 (Thr202/Tyr204) and total ERK1/2 were obtained from Cell Signaling Technology (Beverly, MA). Antibodies to phospho-Smad2 (Ser465/467) were obtained from Millipore (Temecula, CA). Antibodies to total Smad2 were obtained from Zymed (Invitrogen, Carlsbad, CA).

### Odontoblast-enriched preparation from murine teeth

All animal procedures were conducted in agreement with NIH Standards, and approved by the Bioethics Committee of the Faculty of Science, University of Chile, for the care and handling of laboratory animals. Six-week old mice with C57/FVB/N6 genetic background were euthanized and after decapitation, brain and TG tissues were removed and stored at -80°C. Odontoblast-enriched preparations from murine teeth were obtained as previously reported [54]. Briefly, after removal of the upper jaw including the incisors, the gingiva and periodontal connective tissues were dissected along the tooth surface. Connective tissues and blood adhering to the outside of the teeth were carefully removed with a blade and the teeth were suspended in cold saline in a petri dish. Incisors were cleaned with saline and cut from the jaw, rinsed with ice cold saline, and then cut sagitally into two halves. Total RNA was extracted from the pulp and odontoblasts with Trizol (Invitrogen, Carlsbad, CA). Similarly, total RNA was extracted from brain and TG using Trizol.

### Cell culture

Odontoblast-like MDPC-23 cells, derived from rodent dental papilla cells, were kindly provided by Dr. Moon-Il Cho, from the School of Dental Medicine, State University of New York, Buffalo, NY. MDPC-23 cells were cultured in Dulbecco’s modified Eagle’s medium (DMEM, Invitrogen, Carlsbad, CA), supplemented with 10% fetal bovine serum (Hyclone Laboratories, Logan, UT), penicillin, and streptomycin (Invitrogen, Carlsbad, CA) at 5% CO_2_ in a 37°C incubator. MDPC-23 cells were treated in DMEM without serum with DMSO (vehicle), TGF-β1 (1 and 10 ng/ml), SB431542 (0.1, 1, and 10 μM), TGF-β1 (1 ng/ml) plus SB431542 (10 μM), roscovitine (25 μM), and TGF-β1 (1 ng/ml) plus roscovitine (25 μM) at different time points, and total RNA or protein extracts were obtained for qPCR or Western blot analysis, respectively. For differentiation studies, MDPC-23 cells were cultured in complete medium (DMEM plus 10% FBS) supplemented with ascorbate (50 μg/ml) and β-glycerophosphate (10 mM) over 5 days. The medium was changed every 2 days as reported earlier [23].

### RNA isolation

Total RNA using TRIzol^®^ reagent (Invitrogen, Carlsbad, CA) according to the manufacturer’s instructions was obtained from mouse tissues (Odontoblast-enriched preparation, TG, and brain) or from MDPC-23 cells treated with DMSO (vehicle), TGF-β1 (1 ng/ml), SB431542 (10 μM), and TGF-β1 (1 ng/ml) plus SB431542 (10 μM) during 0, 1, 2, and 3 h in serum-free medium. Following TURBO DNA-free (Ambion, Austin, TX) digestion of the total RNA sample, oligo (dT) primed synthesis of cDNA from 3 μg of total RNA was made using SuperScript™ III Reverse Transcriptase (Invitrogen, Carlsbad, CA) to remove contaminated genomic DNA.

### qPCR

For detection of Cdk5, p35, and Egr-1 mRNA from MDPC-23 cells, we used qPCR. The following reaction mixture was used for these PCR samples: 1xIQ™ Sybr^®^Green Super Mix (Bio-Rad, Hercules, CA), 100–200 nM of each primer and 1 μl of cDNA. cDNA was amplified and analyzed in triplicate using Opticon Monitor Chromo 4 (Bio-Rad, Hercules, CA). The following primers were used to amplify and measure the amount of mouse mRNA by qPCR: Cdk5 sense 5′-GGC TAA AAA CCG GGA AAC TC-3′ and antisense 5′-CCA TTG CAG CTG TCG AAA TA-3′; p35 sense 5′-GCC CTT CCT GGT AGA GAG CTG-3′ and antisense 5′-GTG TGA AAT AGT GTG GGT CGG C-3′; Egr-1 sense 5′-CCC TTC CAG GGT CTG GAG AAC CGT-3′ and antisense 5′-GGG GTA CTT GCG CAT GCG GCT GGG-3′. The gene expression level was normalized against S29 expression using these primers: S29 sense 5′-GGA GTC ACC CAC GGA AGT TCG G-3′ and antisense 5′-GGA AGC ACT GGC GGC ACA TG-3′. qPCR samples were run in triplicate each and reaction was repeated three times.

### Conventional RT-PCR

For detection of Cdk5, p35, Tau, DSPP, DMP1, and TRPV1 mRNA from mouse tissues, we used conventional RT-PCR. The reaction mixture was used for these PCR samples: Buffer Taq 10X, MgCl_2_ 2 mM, dNTPs 0.2 mM, and 10 μM of each primer and 4 μl of cDNA. The PCR was performed using Taq (Thermo Scientific) according to the following condition: 5 min hot start at 94°C, 35 cycles of 30 s at 94°C, 30 s at 57-60°C (depending each pair of primers), and 1 min at 72°C, and 10 min at 72°C final extension. The primers used for Cdk5 and p35 were the same that used in PCR. The amplicon size was 228 bp for Cdk5 and 113 bp for p35. Primers for odontoblast markers (DSPP and DMP1) were: DSPP sense 5′-ATT CCG GTT CCC CAG TTA GTA-3′ and DSPP antisense 5′- CTG TTG CTA GTG GTG CTG TT-3′ (108 bp amplicon size); DMP1 sense 5′-GTG CCC AAG ATA CCC CCA G-3′ and antisense 5′-GCA TCC CTT CAT CAT CGA ACT CA-3′ (147 bp amplicon size). Primers for Tau (neuronal marker) were Tau sense 5′- CTG AAG CAC CAG CCA GGA GG-3′and Tau antisense 5′-TGG TCT GTC TTG GGT TTG GC-3′ (368 bp amplicon size). Finally, primers for TRPV1 were TRPV1 sense 5′-CCC TCC AGA CAG AGA CCC TA-3′ and TRPV1 antisense 5′-AGC TGA CGG TGA TGA TAG GG-3′ (171 bp amplicon size). The gene expression level was normalized against S29 expression using primers described above in the qPCR section. PCR products were run in 2% agarose gel.

### Transient transfection

CMV-TRPV1-GFP plasmid (33) was transiently transfected into MDPC-23 cells using Lipofectamine™ LTX and Plus™ Reagent (Invitrogen, Carlsbad, CA) for 48 h. After 24 h of transfection, MDPC-23 cells were treated with TGF-β1 (1 ng/ml), SB431542 (10 μM), and TGF-β1 (1 ng/ml) plus SB431542 (10 μM) over 24 h and then proteins were extracted for Western blot analysis.

### Immunoblot analysis

MDPC-23 cells were treated with DMSO (vehicle), TGF-β1 (1 ng/ml), SB431542 (10 μM), TGF-β1 (1 ng/ml) plus SB431542 (10 μM), TGF-β1 (1 ng/ml) plus U0126 (20 μM) for 0, 1, 2, 3, and 24 h in serum-free medium. MDPC-23 cells were lysed in T-PER buffer (Pierce, Rockford, IL) with protease inhibitor cocktail tablets (Complete Mini) and phosphatase inhibitor cocktail tablets, PhosSTOP (Roche Diagnostic, Indianapolis, IN). Protein concentration in the supernatant was determined using a Bradford Protein Assay (Bio-Rad, Hercules, CA). Proteins were separated in 4-12% or 3-8% SDS-PAGE gels and transferred to nitrocellulose membranes (Invitrogen, Carlsbad, CA). The membranes were soaked in a blocking buffer (5% nonfat dry milk in phosphate-buffered saline with 0.05% Tween-20 (PBST)) for 1 h at room temperature, and then incubated overnight at 4°C with the appropriate primary antibody diluted in the blocking buffer. The membranes were washed in PBST and incubated for 1 h at room temperature with the secondary antibodies diluted in blocking buffer. Immunoreactivity was detected by SuperSignal West Pico or Dura Chemiluminescent Substrate (Thermo Scientific, Rockford, IL). Membranes were stripped for 15 min at room temperature with Re-blot Plus Strong Solution (Chemicon, Temecula, CA) and retested with α-tubulin antibodies to normalize for protein loading. The optical densities of the bands were quantified using an image analysis system with Scion Image Alpha 4.0.3.2 software (Scion Corporation, Frederick, MD).

### Cdk5 kinase activity assay

Cdk5 kinase activity was measured using 250 μg of protein from MDPC-23 cells treated with either vehicle, TGF-β1 (1 and 10 ng/ml), SB431542 (0.1, 1, 10 μM), TGF-β1 (1 ng/ml) plus SB431542 (10 μM), roscovitine (25 μM), or TGF-β1 (1 ng/ml) and roscovitine (25 μM) over 24 h. Proteins were dissolved in T-PER buffer and immunoprecipitated using 4 μg of anti-Cdk5 antibody C8 (Santa Cruz, CA). Immunoprecipitated proteins (IP) were washed 3 times in cold PBS, and 2 times in kinase buffer [20 mM Tris HCl (pH 7.4), 10 mM MgCl_2_ and 1 mM EDTA]. IP were then mixed with the kinase assay mixture [100 mM Tris · HCl (pH 7.4), 50 mM MgCl_2_, 5 mM EDTA, and 5 mM DTT] plus 5 μCi (γP^32^)-ATP, with 5 μg of Histone H1 used as a substrate. Kinase assays were carried out at 30°C for 30 min and the kinase activity reaction was stopped by adding 5xSDS sample buffer and boiling it for 10 min at 70°C. The kinase reaction was electrophoresed on a 4-20% polyacrylamide gel and then gels were exposed to X-ray films for 1–3 h at -80°C. The incorporation of P^32^ to Histone H1 was quantified to measure band intensity using Scion Image Alpha 4.0.3.2 software (Scion Corporation, Frederick, MD).

### MDPC-23 TRPV1 Cell Line

MDPC-23 cells were transfected with rat TRPV1 cDNA in the pϵMTH vector [33] and stable clones were generated after G418 selection (800 μg/ml). Individual clones were screened for TRPV1 activity using calcium imaging and capsaicin stimulation, as described previously [15]. MDPC-23 TRPV1 cells were maintained in high-glucose DMEM (Invitrogen) supplemented with 5% heat-inactivated horse serum (Invitrogen), GlutaMAX (Invitrogen), pen-strep (Invitrogen), Normocin (Invivogen), and G418 (200 μg/ml) to maintain TRPV1 selection. For calcium-uptake assays, cells were plated onto poly-D-lysine coated 96-well plates at a density of 30,000 cells/well. TGF-β1, SB431542, and roscovitine were added after 24 h in culture, when the cells were ~100% confluent. Cells were incubated for an additional 24 h then assayed for TRPV1 activity.

### Assay buffers

The buffer used for ^45^Ca^2+^ uptake assays contained 140 mM NaCl, 5.33 mM KCl, 0.1 mM CaCl_2_, and 2.8 mM MgCl_2,_ and were supplemented with 10 mM Glucose and 26.5 mM Sucrose. The pH was adjusted to 7.4 by the addition of 10 mM HEPES, and 1 mM Ascorbic acid was added to buffers containing capsaicin to prevent oxidation. For ^45^Ca^2+^ uptake assays using proton-rich environments, an unbuffered assay buffer was prepared that contained 140 mM NaCl, 5.33 mM KCl, 0.1 mM CaCl_2_, and 2.8 mM MgCl_2,_ and was supplemented with 10 mM Glucose and 26.5 mM Sucrose (no buffering agent was added). The pH was set to 5.6 by the addition of 15 mM MES hydrate and 5 mM MES Na salt to the unbuffered assay buffer. Lysis buffer was made by diluting stock solutions of 10 M Triton-X-100 and 10 M SDS in ultrapure water to make a final solution containing 1% Triton-X-100 and 1% SDS.

### ^45^Ca^2+^ uptake assay

All ^45^Ca^2+^ uptake assays followed the same protocol and were piloted by a Biomek FX liquid handling robot (Beckman Coulter), which was used in all assays. It was programmed to dilute drugs with ^45^Ca^2+^-containing (0.4 μCi/well) assay buffer on a separate 96-well plate (Evergreen Scientific) in a total volume of 75 μl/well, remove cell culture medium, wash cells with assay buffer, simultaneously transfer drugs and ^45^Ca^2+^ to each well on a 96-well plate, allow for an incubation period of 5 to 8 min. at room temperature, remove ^45^Ca^2+^ and drugs, wash cells with assay buffer, lyse cells in lysis buffer and transfer lysate to a 96-well OptiPlate (white, PerkinElmer Life and Analytical Sciences) preloaded with 125 μl of MicroScint-40 (PerkinElmer Life and Analytical Sciences) scintillation liquid. For manipulations containing assay buffer with a pH of 5.6 or capsaicin, the same protocol was used, however the capsaicin and acidic solutions were preloaded into ^45^Ca^2+^ rich assay buffers to allow for simultaneous transfer. A control capsaicin dose–response dilution was performed on each 96-well plate, along with the treatment, to allow for normalization and to check consistency between experiments. A liquid scintillation counter (TopCount NX, PerkinElmer Life and Analytical Sciences) was used to quantify the ^45^Ca^2+^ signal for further analysis.

### Statistical analysis

All experiments were performed a minimum of 3 times. Statistical evaluation was done with GraphPad Prism software, version 4.0 (GraphPad, San Diego, CA). Significant differences between the experiments were assessed by univariate ANOVA (more than 2 groups) or unpaired t-tests (2 groups). ANOVA was followed by t-tests using a Bonferroni α-correction or Dunnett’s test, where α was set to 0.05.

## Abbreviations

TGF-β1: Transforming growth factor-β1; Cdk5: Cyclin-dependent kinase 5; TRPV1: Transient receptor potential vanilloid-1; Egr-1: Early growth response-1.

## Competing interest

The authors declare no conflict of interest.

## Authors’ contributions

EU, MJI and ABK designed research; EU, MP, AT, JK, JG and ABK performed research; EU, MP, AT, JK, JG, MJI and ABK analyzed data, and EU, JK and ABK wrote the paper. All authors read and approved the final manuscript.

## References

[B1] Magloire H Maurin JC Couble ML Shibukawa Y Tsumura M Thivichon-Prince B Bleicher F Topical review. Dental pain and odontoblasts: facts and hypotheses J Orofac Pain 2010 24 335 349 21197505

[B2] Li Y Lu X Sun X Bai S Li S Shi J Odontoblast-like cell differentiation and dentin formation induced with TGF-beta1 Arch Oral Biol 2011 56 1221 1229 10.1016/j.archoralbio.2011.05.002 21641578

[B3] Vaahtokari A Vainio S Thesleff I Associations between transforming growth factor beta 1 RNA expression and epithelial-mesenchymal interactions during tooth morphogenesis Development 1991 113 985 994 1726565 10.1242/dev.113.3.985

[B4] Thyagarajan T Sreenath T Cho A Wright JT Kulkarni AB Reduced expression of dentin sialophosphoprotein is associated with dysplastic dentin in mice overexpressing transforming growth factor-beta 1 in teeth J Biol Chem 2001 276 11016 11020 10.1074/jbc.M010502200 11116156

[B5] Haruyama N Thyagarajan T Skobe Z Wright JT Septier D Sreenath TL Goldberg M Kulkarni AB Overexpression of transforming growth factor-beta1 in teeth results in detachment of ameloblasts and enamel defects Eur J Oral Sci 2006 114 Suppl 1 30 34 discussion 39–41, 379 16674659 10.1111/j.1600-0722.2006.00276.x

[B6] Kwon SM Kim SA Yoon JH Ahn SG Transforming growth factor beta1-induced heat shock protein 27 activation promotes migration of mouse dental papilla-derived MDPC-23 cells J Endod 2010 36 1332 1335 10.1016/j.joen.2010.04.010 20647091

[B7] He WX Niu ZY Zhao SL Jin WL Gao J Smith AJ TGF-beta activated Smad signalling leads to a Smad3-mediated down-regulation of DSPP in an odontoblast cell line Arch Oral Biol 2004 49 911 918 10.1016/j.archoralbio.2004.05.005 15353247

[B8] Duan X Mao Y Yang T Wen X Wang H Hou J Xue Y Zhang R ClC-5 regulates dentin development through TGF-beta1 pathway Arch Oral Biol 2009 54 1118 1124 10.1016/j.archoralbio.2009.09.008 19878925

[B9] Okumura R Shima K Muramatsu T Nakagawa K Shimono M Suzuki T Magloire H Shibukawa Y The odontoblast as a sensory receptor cell? The expression of TRPV1 (VR-1) channels Arch Histol Cytol 2005 68 251 257 10.1679/aohc.68.251 16477145

[B10] Son AR Yang YM Hong JH Lee SI Shibukawa Y Shin DM Odontoblast TRP channels and thermo/mechanical transmission J Dent Res 2009 88 1014 1019 10.1177/0022034509343413 19828889

[B11] El Karim IA Linden GJ Curtis TM About I McGahon MK Irwin CR Lundy FT Human odontoblasts express functional thermo-sensitive TRP channels: implications for dentin sensitivity Pain 2011 152 2211 2223 10.1016/j.pain.2010.10.016 21168271

[B12] Magloire H Odontoblast and dentin thermal sensitivity Pain 2011 152 2191 2192 10.1016/j.pain.2011.02.042 21377799

[B13] Tsumura M Sobhan U Muramatsu T Sato M Ichikawa H Sahara Y Tazaki M Shibukawa Y TRPV1-mediated calcium signal couples with cannabinoid receptors and sodium-calcium exchangers in rat odontoblasts Cell Calcium 2012 52 124 136 10.1016/j.ceca.2012.05.002 22656960

[B14] Utreras E Terse A Keller J Iadarola MJ Kulkarni AB Resveratrol inhibits Cdk5 activity through regulation of p35 expression Mol Pain 2011 7 49 10.1186/1744-8069-7-49 21736731 PMC3212955

[B15] Utreras E Keller J Terse A Prochazkova M Iadarola MJ Kulkarni AB Transforming growth factor-beta1 regulates Cdk5 activity in primary sensory neurons J Biol Chem 2012 287 16917 16929 10.1074/jbc.M111.329979 22451679 PMC3351291

[B16] Utreras E Futatsugi A Rudrabhatla P Keller J Iadarola MJ Pant HC Kulkarni AB Tumor necrosis factor-alpha regulates cyclin-dependent kinase 5 activity during pain signaling through transcriptional activation of p35 J Biol Chem 2009 284 2275 2284 19049962 10.1074/jbc.M805052200 PMC2629110

[B17] Utreras E Futatsugi A Pareek TK Kulkarni AB Molecular roles of Cdk5 in pain signaling Drug Discov Today Ther Strateg 2009 6 105 111 10.1016/j.ddstr.2009.04.004 21253436 PMC3022326

[B18] Pareek TK Keller J Kesavapany S Pant HC Iadarola MJ Brady RO Kulkarni AB Cyclin-dependent kinase 5 activity regulates pain signaling Proc Natl Acad Sci USA 2006 103 791 796 10.1073/pnas.0510405103 16407116 PMC1325969

[B19] Contreras-Vallejos E Utreras E Gonzalez-Billault C Going out of the brain: non-nervous system physiological and pathological functions of Cdk5 Cell Signal 2012 24 44 52 10.1016/j.cellsig.2011.08.022 21924349

[B20] Arif A Extraneuronal activities and regulatory mechanisms of the atypical cyclin-dependent kinase Cdk5 Biochem Pharmacol 2012 84 985 93 10.1016/j.bcp.2012.06.027 22795893

[B21] Pareek TK Keller J Kesavapany S Agarwal N Kuner R Pant HC Iadarola MJ Brady RO Kulkarni AB Cyclin-dependent kinase 5 modulates nociceptive signaling through direct phosphorylation of transient receptor potential vanilloid 1 Proc Natl Acad Sci USA 2007 104 660 665 10.1073/pnas.0609916104 17194758 PMC1752192

[B22] Terasawa M Shimokawa R Terashima T Ohya K Takagi Y Shimokawa H Expression of dentin matrix protein 1 (DMP1) in nonmineralized tissues J Bone Miner Metab 2004 22 430 438 15316863 10.1007/s00774-004-0504-4

[B23] Hanks CT Sun ZL Fang DN Edwards CA Wataha JC Ritchie HH Butler WT Cloned 3T6 cell line from CD-1 mouse fetal molar dental papillae Connect Tissue Res 1998 37 233 249 10.3109/03008209809002442 9862224

[B24] Hanks CT Fang D Sun Z Edwards CA Butler WT Dentin-specific proteins in MDPC-23 cell line Eur J Oral Sci 1998 106 Suppl 1 260 266 9541235 10.1111/j.1600-0722.1998.tb02185.x

[B25] Sun ZL Fang DN Wu XY Ritchie HH Begue-Kirn C Wataha JC Hanks CT Butler WT Expression of dentin sialoprotein (DSP) and other molecular determinants by a new cell line from dental papillae, MDPC-23 Connect Tissue Res 1998 37 251 261 10.3109/03008209809002443 9862225

[B26] Takahashi S Ohshima T Cho A Sreenath T Iadarola MJ Pant HC Kim Y Nairn AC Brady RO Greengard P Kulkarni AB Increased activity of cyclin-dependent kinase 5 leads to attenuation of cocaine-mediated dopamine signaling Proc Natl Acad Sci USA 2005 102 1737 1742 10.1073/pnas.0409456102 15665076 PMC547862

[B27] Karube H Nishitai G Inageda K Kurosu H Matsuoka M NaF activates MAPKs and induces apoptosis in odontoblast-like cells J Dent Res 2009 88 461 465 10.1177/0022034509334771 19493891

[B28] Yao N Li S Jiang Y Qiu S Tan Y Amelogenin promotes odontoblast-like MDPC-23 cell differentiation via activation of ERK1/2 and p38 MAPK Mol Cell Biochem 2011 355 91 97 10.1007/s11010-011-0842-1 21547453

[B29] Park JH Kwon SM Yoon HE Kim SA Ahn SG Yoon JH Lipopolysaccharide promotes adhesion and migration of murine dental papilla-derived MDPC-23 cells via TLR4 Int J Mol Med 2011 27 277 281 21125213 10.3892/ijmm.2010.568

[B30] Harada T Morooka T Ogawa S Nishida E ERK induces p35, a neuron-specific activator of Cdk5, through induction of Egr1 Nat Cell Biol 2001 3 453 459 10.1038/35074516 11331872

[B31] Holm TM Habashi JP Doyle JJ Bedja D Chen Y van Erp C Lindsay ME Kim D Schoenhoff F Cohn RD Noncanonical TGFbeta signaling contributes to aortic aneurysm progression in Marfan syndrome mice Science 2011 332 358 361 10.1126/science.1192149 21493862 PMC3111087

[B32] Magloire H Couble ML Thivichon-Prince B Maurin JC Bleicher F Odontoblast: a mechano-sensory cell J Exp Zool B Mol Dev Evol 2009 312B 416 424 10.1002/jez.b.21264 19097166

[B33] Olah Z Szabo T Karai L Hough C Fields RD Caudle RM Blumberg PM Iadarola MJ Ligand-induced dynamic membrane changes and cell deletion conferred by vanilloid receptor 1 J Biol Chem 2001 276 11021 11030 10.1074/jbc.M008392200 11124944

[B34] Lin M Luo ZY Bai BF Xu F Lu TJ Fluid mechanics in dentinal microtubules provides mechanistic insights into the difference between hot and cold dental pain PLoS One 2011 6 e18068 10.1371/journal.pone.0018068 21448459 PMC3063177

[B35] Allard B Magloire H Couble ML Maurin JC Bleicher F Voltage-gated sodium channels confer excitability to human odontoblasts: possible role in tooth pain transmission J Biol Chem 2006 281 29002 29010 10.1074/jbc.M601020200 16831873

[B36] Suzuki T Recent progress in sensory mechanism Bull Tokyo Dent Coll 2007 48 1 7 10.2209/tdcpublication.48.1 17721061

[B37] Magloire H Lesage F Couble ML Lazdunski M Bleicher F Expression and localization of TREK-1 K + channels in human odontoblasts J Dent Res 2003 82 542 545 10.1177/154405910308200711 12821716

[B38] Alavi AM Dubyak GR Burnstock G Immunohistochemical evidence for ATP receptors in human dental pulp J Dent Res 2001 80 476 483 10.1177/00220345010800021501 11332536

[B39] Liu X Yu L Wang Q Pelletier J Fausther M Sevigny J Malmstrom HS Dirksen RT Ren YF Expression of ecto-ATPase NTPDase2 in human dental pulp J Dent Res 2012 91 261 267 10.1177/0022034511431582 22173326 PMC3275333

[B40] Pareek TK Kulkarni AB Cdk5: a new player in pain signaling Cell Cycle 2006 5 585 588 10.4161/cc.5.6.2578 16552189

[B41] Yang YR He Y Zhang Y Li Y Han Y Zhu H Wang Y Activation of cyclin-dependent kinase 5 (Cdk5) in primary sensory and dorsal horn neurons by peripheral inflammation contributes to heat hyperalgesia Pain 2007 127 109 120 10.1016/j.pain.2006.08.008 16996690

[B42] Chen HJ Xie WY Hu F Zhang Y Wang J Wang Y Disruption of delta-opioid receptor phosphorylation at Threonine 161 attenuates morphine tolerance in rats with CFA-induced inflammatory hypersensitivity Neurosci Bull 2012 28 182 192 10.1007/s12264-012-1216-8 22466129 PMC5560399

[B43] Xie WY He Y Yang YR Li YF Kang K Xing BM Wang Y Disruption of Cdk5-associated phosphorylation of residue threonine-161 of the delta-opioid receptor: impaired receptor function and attenuated morphine antinociceptive tolerance J Neurosci 2009 29 3551 3564 10.1523/JNEUROSCI.0415-09.2009 19295160 PMC6665260

[B44] Xing BM Yang YR Du JX Chen HJ Qi C Huang ZH Zhang Y Wang Y Cyclin-Dependent Kinase 5 Controls TRPV1 Membrane Trafficking and the Heat Sensitivity of Nociceptors through KIF13B J Neurosci 2012 32 14709 14721 10.1523/JNEUROSCI.1634-12.2012 23077056 PMC6621458

[B45] Li W Fang M Cai XH [Expression and activity of Cdk5/p35 in a rat model of trigeminal neuropathic pain] Shanghai Kou Qiang Yi Xue 2010 19 545 548 21161138

[B46] Rosales JL Lee KY Extraneuronal roles of cyclin-dependent kinase 5 BioEssays 2006 28 1023 1034 10.1002/bies.20473 16998837

[B47] Ishizaki K Takeshima T Fukuhara Y Araki H Nakaso K Kusumi M Nakashima K Increased plasma transforming growth factor-beta1 in migraine Headache 2005 45 1224 1228 10.1111/j.1526-4610.2005.00246.x 16178953

[B48] Bo SH Davidsen EM Gulbrandsen P Dietrichs E Bovim G Stovner LJ White LR Cerebrospinal fluid cytokine levels in migraine, tension-type headache and cervicogenic headache Cephalalgia 2009 29 365 372 10.1111/j.1468-2982.2008.01727.x 19175774

[B49] Piattelli A Rubini C Fioroni M Tripodi D Strocchi R Transforming growth factor-beta 1 (TGF-beta 1) expression in normal healthy pulps and in those with irreversible pulpitis Int Endod J 2004 37 114 119 10.1111/j.0143-2885.2004.00758.x 14871177

[B50] Horst OV Tompkins KA Coats SR Braham PH Darveau RP Dale BA TGF-beta1 Inhibits TLR-mediated odontoblast responses to oral bacteria J Dent Res 2009 88 333 338 10.1177/0022034509334846 19407153 PMC3317952

[B51] Horst OV Horst JA Samudrala R Dale BA Caries induced cytokine network in the odontoblast layer of human teeth BMC Immunol 2011 12 9 10.1186/1471-2172-12-9 21261944 PMC3036664

[B52] Zaremba ML Stokowska W Klimiuk A Daniluk T Rozkiewicz D Cylwik-Rokicka D Waszkiel D Tokajuk G Kierklo A Abdelrazek S Microorganisms in root carious lesions in adults Adv Med Sci 2006 51 Suppl 1 237 240 17460839

[B53] Sloan AJ Perry H Matthews JB Smith AJ Transforming growth factor-beta isoform expression in mature human healthy and carious molar teeth Histochem J 2000 32 247 252 10.1023/A:1004007202404 10872890

[B54] Guo L Berry JE Somerman MJ Davidson RM A novel method to isolate odontoblasts from rat incisor Calcif Tissue Int 2000 66 212 216 10.1007/PL00005837 10666497

